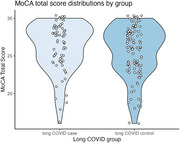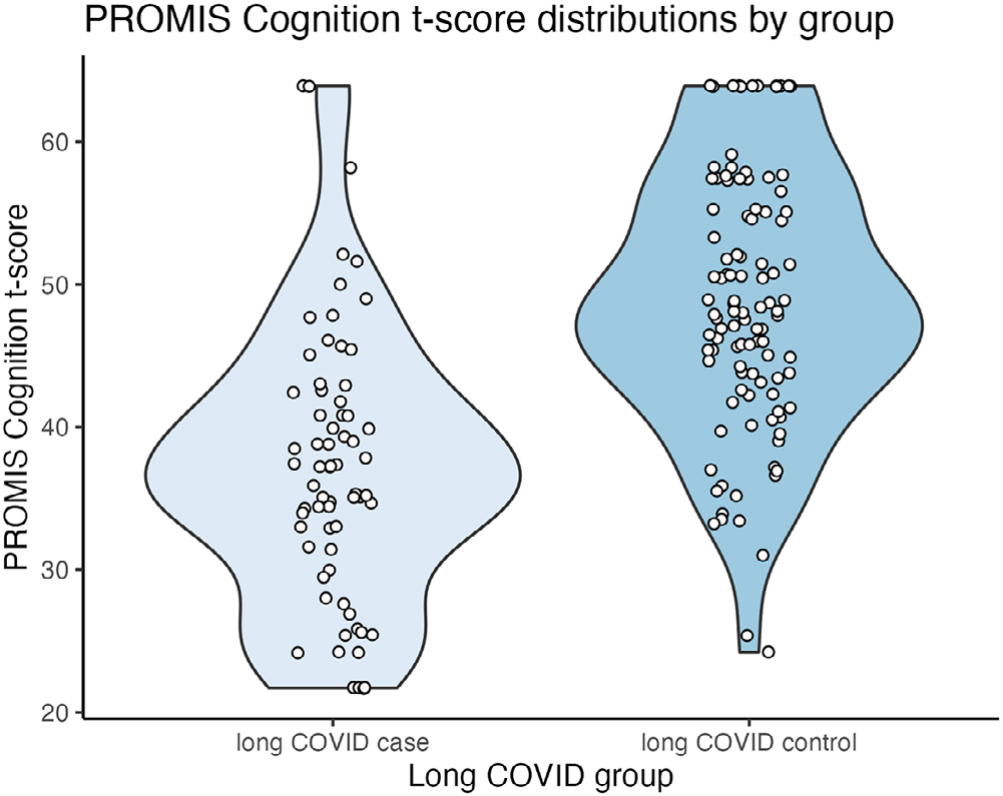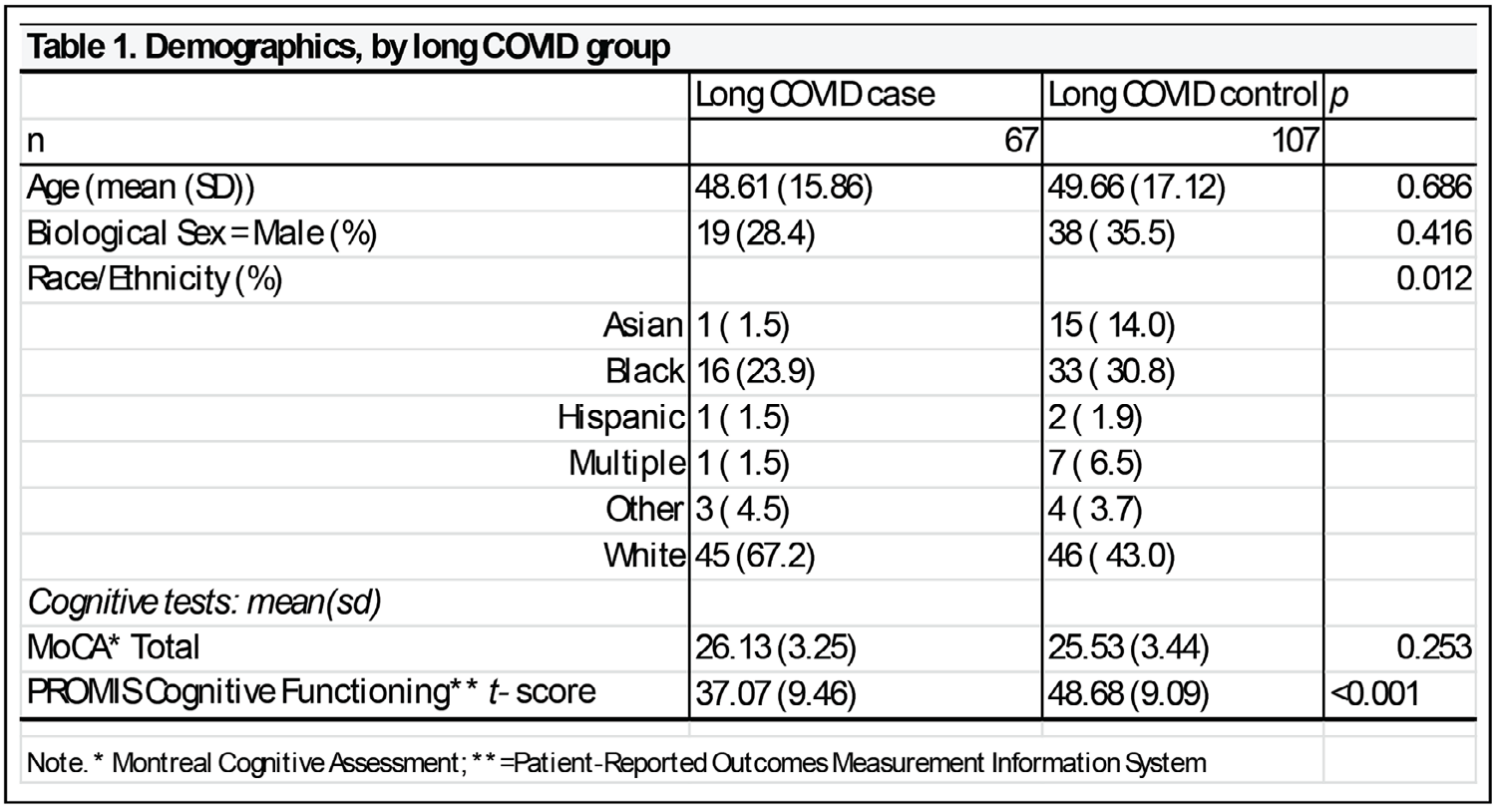# Self‐rated PROMIS cognitive function is a better predictor of long COVID related functional impairment than the Montreal Cognitive Assessment

**DOI:** 10.1002/alz70857_105712

**Published:** 2025-12-26

**Authors:** Kristen E Kehl‐Floberg, Aurora Pop‐Vicas, Dorothy F. Edwards

**Affiliations:** ^1^ University of Wisconsin‐Madison, School of Medicine and Public Health, Madison, WI, USA; ^2^ University of Wisconsin Madison School of Medicine and Public Health, Madison, WI, USA; ^3^ University of Wisconsin Program in Occupational Therapy, Madison, WI, USA

## Abstract

**Background:**

Neurocognitive symptoms are among the most debilitating features of long COVID (persistent, ongoing symptoms and conditions following SARS‐CoV‐2 infection). Traditional neurocognitive screening instruments have been found to be non‐sensitive to post‐COVID cognitive impairments, often described as ‘brain fog’, that disrupt performance of complex daily routines. Accurate screening and diagnosis of cognitive impairment is therefore a critical area of clinical research for people with long COVID. We examined whether self‐reported or neuropsychological cognitive function tests were more strongly associated with long COVID.

**Methods:**

In a sample from a community‐engaged cross‐sectional cohort study, we fitted a binary logistic regression model for the outcome of long COVID status (having history of COVID illness, plus a Post‐COVID Functional Status Scale (PCFS) score >=2), classified by MoCA cut score weighted for race/ethnicity, and associated with the Patient‐Reported Outcomes Measurement Information System (PROMIS) 8‐item Cognitive Functioning t‐scores, controlling for age.

**Results:**

We analyzed data from *N* = 174 participants (*n* = 67 cases, *n* = 107 controls). Our final model was fitted with 5‐knot natural cubic splines for age and PROMIS Cognition scores. At a “Non‐impaired” score on the MoCA, a PROMIS t‐score at the lower quartile of 36.67 increased the odds of having long COVID by over seven times at age 25 (1.0,56.89, *p* = 0.01), over five times at ages 45 (1.4,18.6, *p* = 0.001) and 55 (1.4,20.4, *p* = 0.002), and six times at ages 65 (0.9,51.2, *p* = 0.02) and 75 (0.8,48.9, *p* = 0.03). By contrast, scores above and below race/ethnicity‐weighted MoCA cut score did not significantly affect the odds of having long COVID at any age (*p* value ranges 0.22‐0.25 and 0.19‐0.22, respectively) adjusted for mean PROMIS score.

**Conclusion:**

Across ages, low PROMIS Cognitive Function scores showed between five‐ and six‐fold increases in odds of long COVID, adjusted for age and MoCA score. This self‐rated cognitive function scale was the best predictor of functional status changes attributed to post‐COVID‐19 health changes in our sample.